# Oral Squamous Cell Carcinoma Incidence in Japan Based on National Cancer Registry Data 2016-2019

**DOI:** 10.31662/jmaj.2025-0409

**Published:** 2026-02-20

**Authors:** Shihoko Koyama, Toshitaka Morishima, Kayo Nakata, Nao Nishimura, Miki Ishibashi, Isao Miyashiro

**Affiliations:** 1 Cancer Control Center, Osaka International Cancer Institute, Osaka, Japan; 2 Department of Promoting Cooperation for Community Medicine, Graduate School of Medical Sciences, Nagoya City University, Nagoya, Japan; 3 Department of Oral and Maxillofacial Surgery, Japanese Red Cross Society Kyoto Daini Hospital, Kyoto, Japan; 4 Department of Cancer Oral Care, Dentistry and Oral Maxillofacial Surgery, Osaka International Cancer Institute, Osaka, Japan

**Keywords:** lip and oral cavity cancer, incidence, squamous cell carcinoma

## Abstract

**Introduction:**

No comprehensive national profiles of oral squamous cell carcinoma (OSCC) have been established in Japan. We set out to describe the profiles of OSCC cases, including demographic characteristics, regional disparities, and survival time on a national basis, using a population-based dataset in Japan.

**Methods:**

Using the national cancer registry (NCR) of Japan, we aggregated data on cases of OSCC from 2016 to 2019 that were classified according to the diagnostic criteria specified in the Japanese oral cancer guidelines. We calculated annual, detailed site-specific distributions by sex and age-standardized incidence rates for each year. The standardized incidence ratios by prefecture were computed using 2016 to 2019 data. One-year overall survival was estimated using the Kaplan-Meier method. Univariate and multivariate analyses were conducted using the Cox proportional hazards model.

**Results:**

Between 2016 and 2019, data were obtained on 30,537 OSCC incidence cases. In the most recent year, 2019, 57.0% of patients were men, and the mean age was 70.3 years. Among the specific OSCC sites, the tongue was the most common, accounting for more than half the cases. Over half the cancers were localized at the time of diagnosis. The age-standardized incidence rate in 2019 was 5.12 per 100,000 population. The standardized incidence ratios of OSCC among different prefectures ranged from 0.77 (Gifu Prefecture) to 1.37 (Miyagi Prefecture). The one-year survival rate for all OSCC cases diagnosed between 2016 and 2019 was 83.5%. Cox proportional hazards model analysis, adjusted for all covariates, revealed that women had a significantly lower risk of death within one year than men (hazard ratio = 0.81, 95% confidence interval = 0.76-0.87).

**Conclusions:**

Our study provides an overview of OSCC epidemiology in Japan using the NCR population-based dataset.

## Introduction

Oral cancer is an important global public health problem. According to the latest and most comprehensive analysis reports from GLOBOCAN 2022, 389,485 new cases of lip and oral cancer were diagnosed, making this the 16th most common type of cancer worldwide ^(1)^. At the global level, the age-standardized incidence rate of oral cancer increased slightly from 4.28 per 100,000 in 1990 to 4.52 per 100,000 in 2019. However, the global age-standardized mortality rate from 1990 to 2019 remained stable ^(2)^. In Japan, the age-standardized incidence rate of oral and pharyngeal cancer has increased ^(3)^. Concurrently, a slight increase in the age-standardized mortality rate has been reported by Cancer Statistics ^(4)^.

In Japan, previous studies on oral cancer incidence using population-based cancer registries analyzed one or only a few prefectures until the National Cancer Registry (NCR) was initiated in 2016 ^(5), (6), (7)^. Although the NCR started in 2016, because oral cancer is rare ^(8)^, the cancer incidence reported annually from the National Cancer Center combines oral and pharyngeal cancers. In 2022, the National Cancer Center published the number of oral squamous cell carcinoma (OSCC) cases using 3 years of NCR data in the Rare Cancer Data Book ^(9)^. However, these data have limited detailed information on the anatomical site distribution and sex ratio of OSCC.

The current study was a comprehensive examination to elucidate the incidence of OSCC from data registered between 2016 and 2019 using Japan’s NCR.

## Materials and Methods

### Data

We used data from the population-based NCR in Japan. We received data up to 2019 and used the data from the whole period from 2016 to 2019.

The definition of OSCC sites in this study was based on a previous study by Ota et al. ^(10)^ and followed the International Classification of Diseases for Oncology, 3rd edition (ICD-O-3) site classification: 1. lip (ICD-O-3: C00.0, C00.1, C00.6), 2. buccal mucosa (ICD-O-3: C00.3, C00.4, C06.0, C06.1, C06.2), 3. upper and lower alveolus and gingiva (upper and lower gum) (ICD-O-3: C03.0, C03.1), 4. hard palate (ICD-O-3: C05.0), 5. tongue (ICD-O-3: C02.0, C02.1, C02.2), 6. floor of mouth (ICD-O-3: C04.0). Histological confirmation of squamous cell carcinoma was required (Squamous cell carcinoma 8070/3, Basaloid squamous cell carcinoma 8083/3, Spindle cell squamous cell carcinoma 8074/3, Adenosquamous carcinoma 8560/3, Carcinoma cuniculatum 8051/3, Verrucous squamous cell carcinoma 8051/3, Lymphoepithelial carcinoma 8082/3, Papillary squamous cell carcinoma 8052/3, Acantholytic squamous cell carcinoma 8075/3), as per the classification scheme described in the referenced study ^(9)^.

NCR data include patient-level information on sex, age, detail of specific OSCC site, morphology, survival status (alive or dead), survival time, clinical stage at first treatment in the Surveillance, Epidemiology, and End Results Program staging system (localized/regional/distant/unknown), mode of detection (screening/incidental/other/unknown), and treatment (surgery, radiotherapy, chemotherapy). Regarding the detection mode, screening detection included cases detected by health checkup or cancer screening; incidental detection was defined when diagnosis occurred during follow‐up or surveillance for other diseases. Self-reported symptoms were classified under the ‘Other’ category in the NCR rules.

### Statistical analyses

We collected data on the total number of OSCC cases from the NCR. Using this dataset, we calculated the number of incidences for specific OSCC sites separately for men and women.

Age-standardized incidence rates were analyzed using weighted proportions of corresponding age groups according to the 2015 Japanese standard population ^(11)^.

To compare incidence rates among different prefectures, standardized incidence ratios (SIRs) were calculated using the indirect standardization method by dividing the observed number of OSCC cases by the expected number of cases estimated using the entire Japanese population as a reference.

Survival time was defined as the period from the date of diagnosis to the date of death. The five-year overall survival calculation was limited to data from 2016; thus, one-year overall survival was estimated using the Kaplan-Meier method to utilize a broader range of patient data. Of the 30,537 cases identified between 2016 and 2019, 810 cases were excluded from the survival analysis. These exclusions consisted of 670 cases with a survival time of zero days (diagnosed on the date of death) and 140 cases with duplicate patient identifiers or inconsistent records that could not be processed for survival time calculation. Consequently, 29,727 cases were included in the survival analysis. Univariate and multivariate analyses were conducted using the Cox proportional hazards model. We considered the following as adjustment variables in the statistical model for estimating hazard ratios: age category, sex, detailed site, cancer stage, mode of detection, and treatment.

### Ethical considerations

This study comprised investigative research in accordance with the Cancer Registry Act. We received the registry information in accordance with the law and independently created and processed the provided aggregate/statistical information. According to the rules of the NCR, if there are fewer than 10 cases, the number cannot be explicitly stated for reasons of patient confidentiality. Given that the data were collected under the Cancer Registry Act and fully anonymized, obtaining written informed consent from individual patients was not required.

The study was reviewed and approved by the Research Ethics Committee of the Osaka International Cancer Institute (No.21123-2).

## Results

We confirmed the registration of 30,537 cases of OSCC between 2016 and 2019 (2016: 7,034 cases; 2017: 7,519 cases; 2018: 7,688 cases; 2019: 8,296 cases). Table 1 shows the basic characteristics of OSCC according to sex, age category, specific OSCC site, cancer stage, mode of detection, and treatment by year. Of all cases, 57.2% (17,457 cases) were men, and 52.8% (16,119 cases) were tongue squamous cell carcinoma (SCC). During this period (2016-2019), the average age of all cases, regardless of sex, was 70.3 years (men: 68.3 years; women: 72.9 years). A total of 53.6% of OSCC cases were localized cancer. Less than 1% of cases were detected through screening each year, with more than 75% of cases being discovered through other means, including presentation with symptoms.

**Table 1.** Basic Characteristics of Patients Diagnosed with Oral Squamous Cell Carcinoma (OSCC) by Year (n = 30,537).

		2016		2017		2018		2019		Total	
		N	%	N	%	N	%	N	%	N	%
Total		7,034	100.0%	7,519	100.0%	7,688	100.0%	8,296	100.0%	30,537	100.0%
Sex											
	Men	4,124	58.6%	4,297	57.1%	4,308	56.0%	4,728	57.0%	17,457	57.2%
	Women	2,910	41.4%	3,222	42.9%	3,380	44.0%	3,568	43.0%	13,080	42.8%
Age category											
	-24	26	0.4%	22	0.3%	29	0.4%	35	0.4%	112	0.4%
	25-29	69	1.0%	59	0.8%	39	0.5%	64	0.8%	231	0.8%
	30-34	86	1.2%	92	1.2%	77	1.0%	101	1.2%	356	1.2%
	35-39	120	1.7%	136	1.8%	144	1.9%	132	1.7%	532	1.7%
	40-44	193	2.7%	212	2.8%	227	3.0%	226	2.7%	858	2.8%
	45-49	262	3.7%	276	3.7%	278	3.6%	328	4.0%	1,144	3.7%
	50-54	350	5.0%	354	4.7%	368	4.8%	429	5.2%	1,501	4.9%
	55-59	449	6.4%	463	6.2%	448	5.8%	551	6.6%	1,911	6.3%
	60-64	651	9.3%	659	8.8%	657	8.5%	654	7.9%	2,621	8.6%
	65-69	998	14.2%	1,077	14.3%	965	12.6%	1,032	12.4%	4,072	13.3%
	70-74	947	13.5%	972	12.9%	1,056	13.7%	1,177	14.2%	4,152	13.6%
	75-79	997	14.2%	1,059	14.1%	1,150	15.0%	1,272	15.3%	4,478	14.7%
	80-84	927	13.2%	1,032	13.7%	1,053	13.7%	1,035	12.5%	4,047	13.3%
	85-89	636	9.0%	738	9.8%	772	10.0%	768	9.3%	2,914	9.5%
	90-94	259	3.7%	286	3.8%	333	4.3%	394	4.7%	1,272	4.2%
	95-	64	0.9%	82	1.1%	92	1.2%	98	1.2%	336	1.1%
Mean age		69.8		70.2		70.7		70.3		70.3	
Detailed site											
	Lip	79	1.1%	82	1.1%	91	1.2%	103	1.2%	355	1.2%
	Buccal mucosa	725	10.3%	823	10.9%	843	11.0%	841	10.1%	3,232	10.6%
	Upper and lower gum	2,236	31.8%	2,440	32.5%	2,500	32.5%	2,546	30.7%	9,722	31.8%
	Hard palate	122	1.8%	128	1.7%	143	1.9%	148	1.8%	541	1.8%
	Tongue	3,725	53.0%	3,887	51.7%	3,985	51.8%	4,522	54.5%	16,119	52.8%
	Floor of mouth	147	2.1%	159	2.1%	126	1.6%	136	1.6%	568	1.9%
Cancer stage											
	Localized	3,827	54.4%	4,057	54.0%	3,907	50.8%	4,591	55.3%	16,382	53.6%
	Regional	2,772	39.4%	3,024	40.2%	3,385	44.0%	3,295	39.7%	12,476	40.9%
	Distant	87	1.2%	91	1.2%	104	1.4%	110	1.3%	392	1.3%
	Unknown	348	4.9%	347	4.6%	292	3.8%	300	3.6%	1,287	4.2%
Mode of detection											
	Screening	55	0.8%	38	0.5%	57	0.7%	73	0.9%	223	0.7%
	Incidental	1,487	21.1%	1,606	21.4%	1,643	21.4%	1,788	21.6%	6,524	21.4%
	Other	5,321	75.6%	5,738	76.3%	5,874	76.4%	6,319	76.2%	23,252	76.1%
	Unknown	171	2.4%	137	1.8%	114	1.5%	116	1.4%	538	1.8%
Surgery											
	Yes	5,366	76.3%	5,741	76.4%	5,779	75.2%	6,457	77.8%	23,343	76.4%
	No	1,131	16.1%	1,247	16.6%	1,392	18.1%	1,312	15.8%	5,082	16.6%
	Unknown	537	7.6%	531	7.1%	517	6.7%	527	6.4%	2,112	6.9%
Radiotherapy											
	Yes	1,264	18.0%	1,286	17.1%	1,395	18.1%	1,334	16.1%	5,279	17.3%
	No	5,231	74.4%	5,701	75.8%	5,775	75.1%	6,434	77.6%	23,142	75.8%
	Unknown	539	7.7%	532	7.1%	517	6.7%	528	6.4%	2,116	6.9%
Chemotherapy											
	Yes	1,788	25.4%	1,736	23.1%	1,744	22.7%	1,589	19.2%	6,857	22.5%
	No	4,708	66.9%	5,252	69.8%	5,427	70.6%	6,179	74.5%	21,566	70.6%
	Unknown	538	7.6%	531	7.1%	517	6.7%	528	6.4%	2,114	6.9%

**Figure 1.**
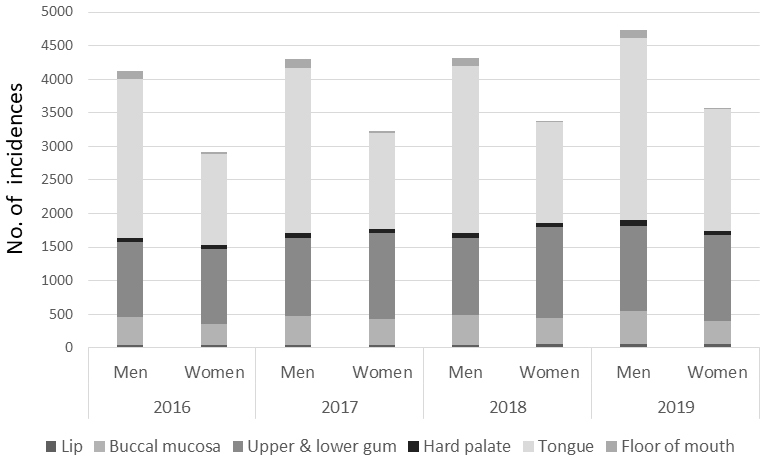
Number of incidences for specific oral squamous cell carcinoma (OSCC) sites separately for men and women.

In the detailed analysis of site-specific numbers of incidence cases stratified by sex, we found there were more cases of lesions on the tongue and floor of mouth among men during this period, while among women there were more cases of lesions on the lips in 2016 and 2018, and the upper and lower gum in 2017-2019 (Figure 1 and Table 2).

**Table 2.** Number of Incidences for Detailed Oral Squamous Cell Carcinoma (OSCC) Sites Separately for Men and Women (n = 30,537).

	Men					Women				
	2016	2017	2018	2019	Total	2016	2017	2018	2019	Total
Lip	38	43	41	53	175	41	39	50	50	180
Buccal mucosa	419	427	450	496	1,792	306	396	393	345	1,440
Upper and lower gum	1,118	1,170	1,143	1,266	4,697	1,118	1,270	1,357	1,280	5,025
Hard palate	64	72	79	85	300	58	56	64	63	241
Tongue	2,358	2,451	2,490	2,710	10,009	1,367	1,436	1,495	1,812	6,110
Floor of mouth	127	134	105	118	484	20	25	21	18	84
Total	4,124	4,297	4,308	4,728	17,457	2,910	3,222	3,380	3,568	13,080

**Figure 2.**
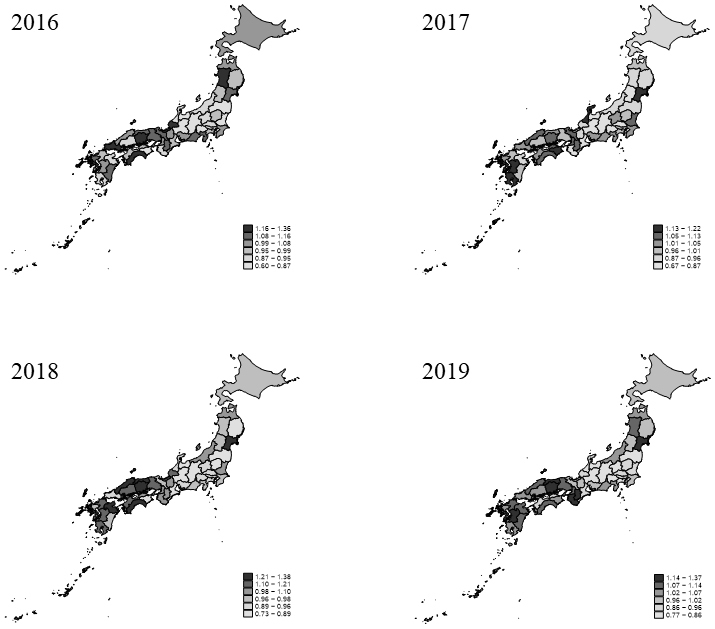
Standardized incidence ratios of oral squamous cell carcinoma (OSCC) in different prefectures in 2016-2019.

Age-standardized OSCC incidence rates were 4.43 per 100,000 population in 2016, 4.70 per 100,000 in 2017, 4.75 per 100,000 in 2018, and 5.12 per 100,000 in 2019, using the 2015 Japanese standard population. The OSCC SIRs among different prefectures in 2019 ranged from 0.77 (Gifu Prefecture) to 1.37 (Miyagi Prefecture; Figure 2).

Table 3 shows the survival and risk of death at one year in OSCC cases (n = 29,727). The one-year survival rate for all OSCC cases diagnosed between 2016 and 2019 was 83.5%. Cox proportional hazards model analysis adjusted for all covariates revealed that women had a significantly lower risk of death within one year than men (hazard ratio [HR] = 0.81, 95% confidence interval [CI] = 0.76-0.87). The oldest age category (95 years or older) had a significantly higher hazard than the youngest category (under 24 years) (HR = 5.72, 95% CI = 2.12-15.45). We focused our analysis on tongue SCC, the most common subtype of OSCC. Compared with tongue SCC, the lip SCCs were associated with a significantly decreased hazard (HR = 0.58, 95% CI = 0.41-0.84). Distant OSCC cases had a significantly higher hazard than those with localized disease (HR = 6.69, 95% CI = 5.72-7.82). The analysis also showed that patients who did not receive treatment had a significantly higher hazard of death within one year than those who did. The hazard was significantly elevated for cases without surgery (HR = 7.12, 95% CI = 6.55-7.75), without radiotherapy (HR = 1.60, 95% CI = 1.48-1.74), and without chemotherapy (HR = 1.66, 95% CI = 1.53-1.80) compared to their respective treated counterparts.

**Table 3.** Survival and Risk of Death at One-Year in Oral Squamous Cell Carcinoma (OSCC) Cases Diagnosed Between 2016 and 2019 (n = 29,727).

	One-year overall survival	Unadjusted analysis	Adjusted analysis*
Total	83.5%	-	-
Sex			
Men	84.7%	Ref	Ref
Women	81.9%	1.20 (1.13-1.27)	0.81 (0.76-0.87)
Age category			
-24	95.5%	Ref	Ref
25-29	96.8%	0.70 (0.20-2.48)	0.72 (0.20-2.53)
30-34	98.4%	0.38 (0.10-1.41)	0.39 (0.10-1.45)
35-39	94.5%	1.22 (0.42-3.52)	1.16 (0.40-3.34)
40-44	96.6%	0.78 (0.27-2.24)	0.71 (0.25-2.04)
45-49	95.2%	1.14 (0.41-3.15)	0.93 (0.34-2.59)
50-54	93.7%	1.44 (0.53-3.94)	1.21 (0.44-3.30)
55-59	91.9%	1.89 (0.70-5.10)	1.37 (0.51-3.71)
60-64	91.3%	2.13 (0.79-5.73)	1.63 (0.60-4.38)
65-69	90.0%	2.40 (0.90-6.43)	1.76 (0.66-4.73)
70-74	87.4%	3.10 (1.16-8.30)	2.18 (0.81-5.83)
75-79	84.3%	3.88 (1.45-10.37)	2.61 (0.98-6.98)
80-84	76.9%	6.10 (2.28-16.28)	3.08 (1.15-8.24)
85-89	64.2%	10.22 (3.83-27.28)	3.82 (1.43-10.21)
90-94	50.2%	15.94 (5.96-42.63)	4.64 (1.73-12.45)
95-	38.1%	23.06 (8.56-62.11)	5.72 (2.12-15.45)
Detailed site			
Lip	90.2%	0.84 (0.58-1.20)	0.58 (0.41-0.84)
Buccal mucosa	81.2%	1.68 (1.53-1.86)	0.90 (0.82-1.00)
Upper & lower gum	76.5%	2.16 (2.02-2.31)	0.89 (0.83-0.95)
Hard palate	77.4%	2.14 (1.75-2.60)	0.91 (0.74-1.11)
Tongue	88.3%	Ref	Ref
Floor of mouth	83.4%	1.44 (1.15-1.80)	0.92 (0.73-1.15)
Cancer stage			
Localized	94.5%	Ref	Ref
Regional	72.7%	5.76 (5.32-6.24)	3.87 (3.55-4.22)
Distant	32.1%	21.15 (18.23-24.53)	6.69 (5.72-7.82)
Unknown	63.3%	8.79 (7.79-9.91)	1.89 (1.64-2.19)
Mode of detection			
Screening	83.8%	Ref	Ref
Incidental	84.5%	1.01 (0.69-1.47)	1.24 (0.85-1.80)
Other	83.3%	1.09 (0.75-1.59)	1.29 (0.89-1.87)
Unknown	79.3%	1.41 (0.93-2.15)	0.81 (0.53-1.24)
Surgery			
Yes	93.4%	Ref	Ref
No	51.5%	10.22 (9.53-10.95)	7.12 (6.55-7.75)
Unknown	49.3%	11.38 (10.45-12.39)	Not Estimable**
Radiotherapy			
Yes	78.4%	Ref	Ref
No	87.7%	0.58 (0.53-0.62)	1.60 (1.48-1.74)
Unknown	49.4%	3.18 (2.91-3.49)	Not Estimable**
Chemotherapy			
Yes	82.2%	Ref	Ref
No	87.2%	0.73 (0.68-0.79)	1.66 (1.53-1.80)
Unknown	49.3%	3.89 (3.56-4.25)	Not Estimable**

Ref: reference. *Adjusted for sex, age category, detailed site, cancer stage, mode of detection, surgery, radiotherapy, and chemotherapy. **Unknown for Surgery, Radiotherapy, and Chemotherapy variables resulted in Complete Separation during Cox regression. This prevented estimation of the hazard ratio and 95% confidence interval (not estimable). Complete separation occurs when all subjects in a category either experience or do not experience the event.

## Discussion

To our knowledge, the current study is the first to characterize OSCC profiles, including incidence and survival, on a national basis in Japan, using population-based data. In particular, given the rarity of OSCC, there has been a paucity of studies investigating the annual incidence of OSCC by detailed subsite and sex.

Although the exact definition of oral cancer in a previous (1999) study is unclear, it predicted that there would be 7,800 cases in 2015 ^(12)^. However, our data show that OSCC incidence in 2016 was lower than the previous study figure but subsequently increased, exceeding 7,800 cases by 2019. One possible explanation for the surge in cases in 2018 and 2019 could be the heightened public awareness when a high-profile Japanese entertainer was diagnosed with tongue cancer ^(13)^.

Previous studies have consistently reported a higher prevalence of oral cancer in males than in females, with a male-to-female ratio of approximately 3:2 ^(14)^. Our finding is consistent with these previous reports, also showing a male-to-female ratio of 3:2 for oral cancer. Our results indicate that the gender difference in the number of OSCC cases is primarily attributed to the disparity in the number of cases of tongue and floor of mouth SCC. This higher incidence in men may be attributable to the fact that more men than women smoke and to the increased exposure to harmful substances when smoking, particularly in sites such as the tongue, floor of the mouth, and buccal mucosa. Meanwhile, we found more incidences of upper and lower gum SCC among women than men. While smoking rates have been decreasing in both sexes, alcohol consumption has remained relatively stable in men while increasing in women ^(15)^. Therefore, the sex ratio of OSCC incidence may shift in the future. Regarding the prognosis of OSCC, Table 3 shows that the unadjusted HR for women was 1.20 (indicating a higher risk of death than men). However, multivariate adjustment resulted in a significantly lower HR of 0.81. This notable reversal is primarily attributed to the significant disparity in age distribution (women: 72.9 years; men: 68.3 years). Specifically, the inclusion of age as a covariate accounted for the majority of the observed attenuation and reversal of the HR, suggesting that the initial difference was largely due to the women’s older age.

The SIRs varied across prefectures, from the lowest at 0.77 to the highest at 1.37 in 2019. Overall, western prefectures showed higher SIRs. However, the distribution of risk factors for oral cancer, such as smoking and alcohol consumption rates, and the prevalence of dental caries, was worse in northern prefectures ^(15), (16)^, contrasting with the distribution of OSCC cases. In Japan, prosthetic treatments are relatively inexpensive due to the universal health insurance system ^(17)^. The use of dentures and mechanical irritation from ill-fitting prostheses are considered causes of oral cancer ^(18)^. Previous studies have reported a higher supply of tooth extraction treatment and occlusal restorative treatments in western Japan, suggesting a potential association ^(19)^.

The one-year survival rate for all OSCC cases diagnosed between 2016 and 2019 was 83.5%. The National Cancer Center of Japan reported a five-year relative survival rate of 63.5% for oral and oropharyngeal cancer; the five-year relative survival rate varied significantly based on clinical stage: 86.6% for localized stage, 53.5% for regional stage, and 13.9% for distant stage ^(4)^. Our findings align with these results, demonstrating a similar trend. Specifically, the pronounced staging gradient (where survival decreases markedly as the disease progresses from localized to regional and then to distant stage) is consistent between our one-year overall survival data and the established five-year rates.

Multivariate analyses showed a significant association between advanced age and advanced cancer stage with increased one-year mortality. Approximately 50% of the patients had localized disease, while the remaining 50% had advanced disease. The observed staging gradient in one-year overall survival―where survival decreases sharply with progression from localized to distant disease―is consistent with established principles in head and neck oncology. Cancer stage remains the single most impactful prognostic determinant, primarily because the presence of regional lymph node metastasis signifies a critical shift in tumor biology, indicating a higher systemic disease burden and potential for distant spread ^(20)^. A previous study showed that patients who had a regular primary care dentist were more likely to be diagnosed at early stages than those who did not ^(21)^. In addition, another previous meta-analysis indicated a significant association between lack of dental visits (never/irregular/not frequent) and incidence of head and neck cancers, particularly for oral cancers ^(22)^. The planned implementation of nationwide dental checkups in Japan in 2025 may potentially enhance the early detection of OSCC and improve patient outcomes.

The results of our Cox proportional hazards model clearly demonstrated that the hazard was significantly and substantially higher for patients who did not receive treatment, whether it be surgery, radiotherapy, or chemotherapy. The extremely high hazard ratio (HR = 7.12) observed in the non-surgical group is particularly noteworthy, strongly suggesting that resection of the tumor is crucial for survival in OSCC. This finding aligns with previous research indicating that multimodality treatment significantly enhances the five-year survival rate for OSCC patients ^(23)^.

The present study has some limitations. First, data collection for the NCR database used in this study began in 2016. Therefore, it was not possible to conduct long-term survival analyses or detailed subgroup analyses of OSCC-related deaths. We anticipate that further data collection will enable such analyses in the future. Second, the NCR dataset does not provide detailed patient information, such as oral conditions and granular details of surgical procedures. Oral hygiene factors such as the number of remaining teeth, prosthetics, and daily oral hygiene habits are believed to influence both OSCC incidence and mortality. The NCR dataset lacks oral hygiene-related detail, which prevented us from incorporating these factors into our analysis. We would like to examine the association between oral condition and OSCC incidence, considering details of treatment, using the oral cancer registry initiated by the Japan Society of Oral and Maxillofacial Surgery for future analyses.

Despite these limitations, we believe that the present study is valuable because it is the first to characterize OSCC profiles, including demographic characteristics, regional disparities, and survival time, on a national basis using a population-based dataset in Japan.

This study provides a comprehensive overview of OSCC epidemiology in Japan using NCR data. Our findings represent the first nationwide characterization of OSCC profiles based on a population-based database in Japan.

## Article Information

### Acknowledgments

This study was supported by JSPS (Japan Society for the Promotion of Science) KAKENHI Grant Number (JP22K17280, JP24K13129). The funders had no role in the study design, data collection, or analysis. The views and opinions expressed in this article are those of the authors and do not necessarily reflect the official policy or position of the respective funding organizations.

We thank Dr. Julia Mortimer for her assistance with the English language editing of the manuscript, and Gemini, a large language model, for its help with the English language translation.

### Author Contributions

Conceptualization: Shihoko Koyama; methodology: Shihoko Koyama; software: Shihoko Koyama; validation: all authors; formal analysis: Shihoko Koyama; investigation: Shihoko Koyama; resources: Shihoko Koyama; data curation: Shihoko Koyama; writing―original draft preparation: Shihoko Koyama; writing―review and editing: Shihoko Koyama, Toshitaka Morishima, Kayo Nakata, Nao Nishimura, Miki Ishibashi, and Isao Miyashiro; visualization: Shihoko Koyama; supervision: Toshitaka Morishima and Miki Ishibashi; project administration: Shihoko Koyama; funding acquisition: Shihoko Koyama. All authors have read and agreed to the published version of the manuscript.

### Conflicts of Interest

Dr. Shihoko Koyama is affiliated with the Department of Promoting Cooperation for Community Medicine, an endowed chair supported by the City of Gamagori.

### IRB Approval Code and Name of the Institution

The study was reviewed and approved by the Research Ethics Committee of the Osaka International Cancer Institute (No.21123-2).

### Data Availability

The data that support the findings of this study are available on request from the corresponding author. The data are not publicly available due to privacy or ethical restrictions.
